# The relation between staging fluorine-18 fluorodeoxyglucose positron emission tomography/computed tomography metabolic parameters and tumor necrosis rate in pediatric osteosarcoma patients

**DOI:** 10.3906/sag-2004-358

**Published:** 2021-06-28

**Authors:** Aykut Kürşat FİDAN, Gülin UÇMAK, Bedriye Büşra DEMİREL, Hülya EFETÜRK, İpek ÖZTÜRK, Semra DEMİRTAŞ ŞENLİK2, Burcu ESEN AKKAŞ, İnci ERGÜRHAN İLHAN

**Affiliations:** 1 Department of Nuclear Medicine, Hitit University Çorum Erol Olçok Education and Training Hospital, Çorum Turkey; 2 Department of Nuclear Medicine, University of Health Sciences Dr. Abdurrahman Yurtaslan Ankara Oncology Education and Training Hospital, Ankara Turkey; 3 Department of Nuclear Medicine, Mehmet Akif İnan Research and Training Hospital, Şanlıurfa Turkey; 4 Department of Nuclear Medicine, University of Health Sciences Sultan Abdulhamid Han Education and Training Hospital, İstanbul Turkey; 5 Department of Pediatric Hematology-Oncology, University of Health Sciences Dr. Abdurrahman Yurtaslan Ankara Oncology Education and Training Hospital, Ankara Turkey

**Keywords:** Pediatric osteosarcoma, positron emission tomography, tumor necrosis rate

## Abstract

**Background/aim:**

The aim of this study was to investigate the contribution of fluorine-18 (F-18) fluorodeoxyglucose (FDG) positron emission tomography/computed tomography (PET/CT) imaging in staging of pediatric osteosarcoma patients and also to evaluate the ability of metabolic parameters from the primary tumor to predict tumor necrosis rate (TNR).

**Material and methods:**

F-18 FDG-PET/CT imaging was performed in staging 37 pediatric osteosarcoma patients. The metabolic parameters SUVmax (maximum standardised uptake value), MTV (metabolic tumour volume), and TLG (total lesion glycolysis) were measured from the primary tumor. TNR level of the primary tumor was histopathologically measured after standard neoadjuvant chemotherapy treatment. The contribution of F-18 FDG-PET/CT to staging of pediatric osteosarcoma patients and the accuracy of metabolic parameters of the primary tumor to predict TNR were analized by regression analysis.

**Results:**

MTV and TLG of the primary tumor were found to efficiently predict histopathologic TNR, whereas SUVmax was not (P = 0.012, P = 0.027, P = 0.25, respectively). Also 5 of 12 patients (41.6%) who were initially defined as localised osteosarcoma were upstaged in consequence of staging F-18 FDG-PET/CT findings.

**Conclusion:**

F-18 FDG-PET/CT staging in pediatric osteosarcoma patients can effectively distinguish metastatic-localised disease. MTV and TLG values are important parameters, which can efficiently be used to predict TNR.

## 1. Introdution: 

The most common primary malignant bone tumor in children and young adolescents is osteosarcoma, which is generated from mesenchymal stem cells taking part in bone formation [1]. Metastatic dissemination is common at the time of diagnosis and follow-up; about 80% disseminates to the lungs and 20% is detected at the time of diagnosis and is rarely observed in the bone and lymph nodes. 5-year survival is around 65%–70% in localised disease and decrease to 20% in the presence of metastasis [1,2]. Correct staging of osteosarcoma at the time of presentation plays a crucial role in the choice of chemotherapy protocol and additional therapies, such as radiotherapy and radionuclide therapy.

Prognostic markers used in osteosarcoma are age, sex, localisation of tumor, size of tumor, histologic grade, stage of disease, level of alkaline phosphatase (ALP) and lactate dehydrogenase (LDH), and histopathologic tumor necrosis rate (TNR) after neoadjuvant therapy. TNR in osteosarcoma is a parameter indicating the degree of necrotic tissue of the excised primary tumor post neoadjuvant chemotherapy and has more prognostic importance in comparison with the other markers. It is well known that in patients with higher TNR, disease-free survival is longer compared to those with a lower TNR; thus, adjuvant chemotherapy protocols can be adjusted according to the degree of TNR [3]. It has been shown in a variety of studies that fluorine-18 (F-18) fluorodeoxyglucose (FDG) positron emission tomography/computed tomography (PET/CT) imaging modality has a higher sensitivity (98% versus 83%) and specificity (97% versus 73%) in detecting distant metastasis other than lung metastasis in primary malignant bone tumors [4]. While F-18 FDG-PET/CT and bone scintigraphy have similar sensitivity and spesificity in detection of bone metastases in osteosarcoma, the combination of the two modalities can yield a higher sensitivity when used together [5]. 

F-18 FDG-PET/CT metabolic parameters, such as peak tumoral activity uptake known as standardised maximum uptake value (SUVmax), mean value (SUVmean), metabolic tumor volume of viable tumor tissue (MTV), and total lesion glycolysis (TLG) used in evaluating metabolism, can be measured semiquantitatively. The importance of SUVmax and TLG values calculated from F-18 FDG-PET/CT performed before and after chemotherapy has been highlighted as important prognostic indicators of progression-free and overall survival in osteosarcoma patients [6]. Metabolic parameters of the primary tumor in staging F-18 FDG-PET/CT has been shown to be important prognostic markers foreseeing response to neoadjuvant chemotherapy and TNR in the limited number of studies carried out [6].

This study aimed to review retrospectively the contribution of F-18 FDG-PET/CT findings to staging and the value of metabolic parameters of the primary tumor to predict TNR in pediatric osteosarcoma patients staged with F-18 FDG-PET/CT.

## 2. Material and methods

### 2.1. Patients selection

After formal institutional review board and local educational planning board approval (July 21, 2016) by the University of Health Sciences Dr. Abdurrahman Yurtaslan Ankara Oncology Education and Training Hospital, Ankara, Turkey, a retrospective study was conducted on a total of 37 patients from our center in the pediatric age group (6-18 years) with a histologic diagnosis of osteosarcoma evaluated for staging/extent of disease using F-18 FDG-PET/CT between June 2009 and December 2016. Data collected included age of diagnosis, histological subtype, tumor size, location of primary disease, presence of metastatic disease at diagnosis, histopathologic findings including TNR at resection, radiologic, scintigraphic, laboratory findings, and neoadjuvant chemotherapy protocols.

### 2.2. Image review and tumor characteristics 

All patients fasted for at least 6 h and had a blood glucose level below 150 mg/dL. After being injected with 0.09–0.12 mCi/kg intravenous F-18 FDG followed by 60 min of rest, a whole body scan in supine position was carried out. Dual-phase imaging and/or additional images were obtained from selected patients in case of suspicion or necessity. All patients were evaluated using three-dimensional Siemens Biograph True Point 6 PET/CT device. The PET scanner and 3 mm sliced multidetector CT scanner obtained simultaneous images in the same session. Low dose CT images without intravenous iodinated contrast were used for attenuation-correction and anatomy-correlation. All PET/CT images were reconstructed and reviewed on Siemens Leonardo PET/CT workstation. All metabolic parameters were taken from a volume of interest (VOI) drawn around the primary tumoral hypermetabolic lesion with a SUV-based automatic contouring programme. Lesion contours were checked for neighboring normal tissue by looking at axial, coronal, and sagittal sections and manually reshaping the VOI. The SUVmax value in the volume of interest was recorded and a threshold SUV of 2.0 mg/dL was used to automatically measure MTV from the metabolically active tumor in the VOI. TLG values were measured as a product of SUVmean and MTV. Metabolic parameters from lesions suspicious for metastasis were also recorded.

Histological responses to preoperative chemotherapy were assessed for operated tumor tissues, and all the tumoral tissue including the largest cross section were ascertained to evaluate the response to chemotherapy by pathologist in our center. The response was evaluated based on percentage of tumor necrosis or viable tumor cells as follows: good responders had equal to or greater than 90% tumor necrosis or less than 10% viable tumor cells, on the other hand, poor responders had less than 90% tumor necrosis or greater than 10% viable tumor cells based on histopathological reports [7,8].

Patients were classified as localised disease (stage 1 and 2) and metastatic disease (stage 3 and 4) in osteosarcoma with respect to the American Joint Committee on Cancer (AJCC) criteria (Table 1). Lesions evaluated as metastasis/suspicious for metastasis found on F-18 FDG-PET/CT or other imaging modalities (radiologic imaging, bone scintigraphy) were either histopathologically verified or reevaluated and decided upon after 6-month clinical, metabolic and morphological follow-up by means of nuclear and radiology imaging modalities. Findings obtained from conventional radiologic and nuclear imaging modalities as well as additional information obtained from staging F-18 FDG-PET/CT were recorded. 

**Table 1:** Staging according to AJCC criteria in extremity osteosarcoma.

Stage	Tumor grade	Tumor size	Metastasis
1A	Low	≤ 8 cm	None
1B	Low	> 8 cm	None
2A	High	≤ 8 cm	None
2B	High	> 8 cm	None
3	Low & high	Any size	Skipped metastasis
4A	Low & high	Any size	Lung metastasis
4B	Low & high	Any size	Extrapulmonary / lymphatic metastasis

### 2.3. Statistical analysis

TNR level is one of the most independent prognostic indicators evaluated in pediatric osteosarcoma. Prediction of TNR levels under standard therapy will provide important information in determining the prognosis of the disease. In addition, the presence of metastases and the region of metastases are parameters that should not be ignored in determining the prognosis. The metabolic parameters of the staging F-18 FDG-PET/CT imaging parameters (SUVmax, MTV, TLG values) ability to predict lung, lymphatic, bone and bone marrow metastases and postchemotherapy TNR levels (categorized higher and lower than 90%) were determined by logistic regression analysis. The logistic regression model was used to estimate the odds ratio (OR) in univariate analysis. Median values were used as cut-off values for covariates that were not normally distributed. Statistical tests were performed using Statistical Package for the Social Sciences (SPSS) software version 15.0 (IBM Corp., Chicago, IL, USA). Statistical significance was set at the 5% level. 

## 3. Results

### 3.1. Patient characteristics, demographic and clinical data

37 eligible patients (21 male (56%) and 16 female (44%)) between the ages 6–18 (mean 14) with histopathologically proven diagnosis of osteosarcoma were enrolled in this study (Table 2). Histopathological subtypes of 37 patient’s preoperative biopsy was high grade conventional osteosarcoma (osteoblastic and chondroblastic osteosarcoma) in 34 of 37 patients (91.9%), parosteal osteosarcoma in 2 patients, one of which was high grade (5.4%) and low grade periosteal osteosarcoma in 1 patient (2.7%). The location of the primary tumor was the femur in 24 patients (64.9%), the tibia in 6 patients (16.2%), the fibula in 3 patients (8.1%), and the humerus in 4 patients (10.8%). 

**Table 2:** Patient characteristics.

Characteristic		Values
Age, median (range)		14(6–18)
Sex	Male	21 (56%)
	Female	16 (44%)
Primary tumor localization	Femoral	24 (64.9%)
	Tibial	6 (16.2%)
	Fibular	3 (8.1%)
	Humeral	4 (10.8%)
Histopathological type	Conventional (High grade)	2 (5.4%)
	Parosteal	34 (91.9%)
	Periosteal	1 (2.7%)
Stage	Localised (Stage 1A-B, 2A-B)	17 (46%)
	Metastatic (Stage 4A-B)	20 (54%)
Tumor necrosis rate	< 90%	18 (53%)
	≥ 90%	15 (44%)
	Unknown	1 (3%)

AJCC stages were as follows: 2 patients stage 1 (5.4%), 2 patients stage 2A (5.4%), 13 patients stage 2B (35.1), 7 patients stage 4A (18.9) and 13 patients stage 4B (35.1%). At the time of initial staging, 17 patients (45.9%) were free of distant metastasis and 20 patients (54.1%) had distant metastasis. Of the 20 patients with distant metastasis 7 patients (35%) had lung metastasis, 2 of them also had intramedullary skip metastasis; 6 patients (30%) had lymphatic metastasis, 2 of them also had intramedullary skip metastasis; 4 patients (20%) had both lung and lymphatic metastasis, 1 of them also had intramedullary skip metastasis; 3 patients (15%) had bone, lung and lymphatic metastasis, 1 of them also had intramedullary skip metastasis.

In 12 patients, F-18 FDG-PET/CT used for staging purposes detected metastatic lymph nodes in addition to detecting all findings of other radiological modalities performed (magnetic resonance imaging (MRI) of primary tumor, CT evaluation of the lung), hence upstaging 5 patients (41.6%) from localised to metastatic disease status. Staging bone scan revealed osteoblastic bone metastasis in 1 of 3 patients with metastatic disease and lung metastasis on diagnostic thorax CT evaluation. In 2 patients, F-18 FDG-PET/CT scan additionally revealed lytic bone metastasis, which was not visible on bone scan, contributing to treatment plan and follow-up in these patients. 36 of 37 patients included in this study were treated with neoadjuvant chemotherapy, 34 patients were operated (24 patients (64.9%) went through limb-sparing surgical procedure, 10 patients (27%) were amputated) and 3 patients were deemed inoperable due to advanced disease. 

Postoperative histopathological analysis of 33 patients was a follows: The histological subtype was high grade conventional osteosarcoma (osteoblastic and chondroblastic osteosarcoma) in 30 patients, parosteal osteosarcoma in 2 patients, one of which was high grade and periosteal osteosarcoma in 1 patient. The postoperative histopathology evaluation of 1 patient was not reviewed due to being operated in another center, hence was excluded from the study. Tumor necrosis rate on postoperative specimen was of low necrosis degree (< 90%) in 18/33 patients (55%) and high necrosis degree (≥ 90%) in 15/33 patients (45%). 

### 3.2. The relationship between F-18 FDG-PET/CT metabolic parameters (SUVmax, MTV, TLG) and TNR 

The median SUVmax value of the primary tumor in all patients was 9.7 mg/dL, median MTV value was 249.5 mL and median TLG value was calculated as 1074 gr. When the median value was used as a cut-off value, logistic regression analysis used to analyse the ability of these metabolic parameters to predict tumor necrosis rate after standard chemotherapy revealed that high SUVmax values could not predict TNR (P = 0.25), but patients with higher MTV and TLG values than the cut-off value were found to have low (< 90%) degree of TNR (P = 0.012, P = 0.027, respectively). Patients with low degree of TNR had a median SUVmax value of 10.08 mg/dL, median MTV value of 399 mL and median TLG value of 1648 gr, while patients with high level (≥ 90%) TNR had a median SUVmax value of 9.18 mg/dL, median MTV value of 130 mLand median TLG value of 605 gr. As can be seen in one of the our cases we used in this study; the case is localised osteosarcoma patient whose primary tumor on right distal femur (Figure). The tumor is visible on maximal density image (Figure, a) and sectional views (Figure, b) in F-18 FDG- PET/CT scan. The tumor has lower MTV (99.7 mL), TLG (399.7 gr) and higher SUVmax (10.34 mg/dL) levels than measured median PET metabolic parameter levels with VOI method in F-18 FDG-PET/CT scan (Figure, b). TNR of the patient’s tumoral lesion is 100% (higher than 90%) after neoadjuvant therapy.

**Figure:**
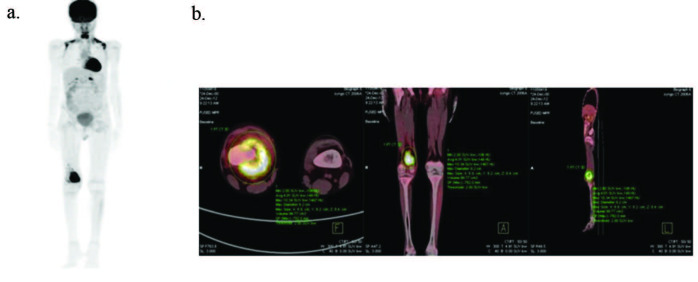
Localised osteosarcoma patient (12-y, m) with low metabolic parameters (MTV and TLG) of primary tumor. On staging F-18 FDG-PET/CT images; primary tumor on right distal femur: Maximal density image (a). VOI from the primary tumor (b), taken from the axial, coronal, sagittal sections and measured metabolic parameters; SUVmax: 10.34 mg/dL, MTV: 99.7 mL, TLG: 399.7 gr. The patient, whose TNR was 100% after neoadjuvant therapy, has been followed up for 49 months without disease. F-18 FDG-PET/CT: Fluorine-18 fluorodeoxyglucose positron emission tomography/computed tomography; SUVmax: Maximum standardised uptake value; MTV: Metabolic tumor volume; TLG: Total lesion glycolysis; VOI: Volume of interest; TNR: Tumor necrosis rate.

Unlike MTV and TLG, factors used in prognostification of pediatric osteosarcoma patients such as age of diagnosis, location of tumor, level of LDH-ALP, tumor grade, tumor size, solid organ or lymphatic metastasis, and sex were not found to predict post-therapy TNR (Table 3).

**Table 3:** Parameters that predict TNR levels below 90% with univariate logistic regression analysis.

Parameters	High TNR (n = 15)	Low TNR(n = 18)	OR (CI, 95%)	P-value
SUVmax	9.18 mg/dL	10.08 mg/dL	1.02 (0.89–1.17)	0.25
MTV	130 mL	399 mL	7.10 (1.5–33.3)	0.012*
TLG	605 gr	1648 gr	5.52 (1.2–24.8)	0.027*
Sex	-	-	0.71 (0.17–2.77)	0.61
Age	12	15	1.19 (0.96–1.47)	0.3
Region of tumor	-	-	0.81 (0.09–7.01)	0.32
LDH	266 u/L	365 u/L	0.99 (0.98–1.003)	0.79
ALP	243 u/L	469.5 u/L	1.004 (1.00–1.008)	0.08
Metastasis	-	-	4.00 (0.93–17.11)	0.06
Tumor grade	-	-	0.82 (0.004–17.38)	0.82

### 3.3. The relationship between F-18 FDG-PET/CT metabolic parameters (SUVmax, MTV, TLG) and regional metastasis

When the median value was used as a cut-off value; in patients with MTV and TLG values higher than cut-off values, there was a greater possibility of detecting lung and lymphatic metastasis at staging. On the other hand, these values were not able to predict the presence of bone (P = 0.17, P = 0.08, respectively) and bone-marrow metastasis (P = 0.09, P = 0.08, respectively). Similarly, the SUVmax value of the primary tumor was not able to predict lung, lymphatic, bone, or bone-marrow metastasis (P = 0.25, P = 0.14, P = 0.27, P = 0.45, respectively) (Table 4). 

**Table 4:** Regional metastasis prediction level of PET metabolic parameters.

Region of metastasis		SUVmax	MTV	TLG
Lung	met. (+) n = 15	13.3 mg/dL	400 mL	1930 gr
	met. (–) n = 22	8.9 mg/dL	137 mL	618 gr
	OR (CI, 95%)	0.82 (0.78–1.12)	2.32 (1.35–5.94)	1.86 (1.12–3.64)
	p-value	0.25	0.001*	0.001*
Lymphatic	met. (+) n = 12	13.8 mg/dL	458 mL	2163 gr
	met. (–) n = 25	9.18 mg/dL	157 mL	716 gr
	OR (CI, 95%)	0.79 (0.71–1.24)	2.17 (1.23–4.67)	1.79 (1.08–3.44)
	p-value	0.14	0.001*	0.001*
Bone marrow	met. (+) n = 6	13 mg/dL	447 mL	1878 gr
	met. (–) n = 31	9.7 mg/dL	228 mL	1057 gr
	OR (CI, 95%)	1.06 (0.92–1.18)	0.85 (0.67–1.18)	0.96 (0.80–1.31)
	p–value	0.45	0.09	0.08
Bone	met. (+) n = 3	13.9 mg/dL	394 mL	1930 gr
	met. (–) n = 34	9.67 mg/dL	238 mL	1140 gr
	OR (CI, 95%)	0.89 (0.76–1.12)	1.02 (0.97–1.43)	0.74 (0.70–1.27)
	p-value	0.27	0.17	0.08

## 4. Discussion

Osteosarcoma is the most common primary malignant bone tumor occuring in pediatric age group and young adults. Metastatic disease is one of its most important prognostic markers. While 5-year overall survival is around 65%–70% in nonmetastatic localised disease, it can fall to 20% in the presence of metastatic disease. Metastatic dissemination is a common finding at time of diagnosis and follow-up, also it occurs in lungs by 80% (20% at time of diagnosis) and rarely to the bones and lymph nodes [1,2]. With the development of chemotherapy protocols and targeted treatment, there has been a significant increase in progression-free and overall survival in comparison to previous rates. Other important prognostic factors are the tumor location, tumor size, patient age, histologic subtype, AJCC stage, site of metastasis, extent of surgery/type of surgery, presence of tumor at surgical margin, and histologic response to chemotherapy [9]. 

Metastasis at time of diagnosis considerably decreases progression-free and overall survival time making significant changes in the treatment plan. Accurate staging of disease has a major effect on the selection of neoadjuvant chemotherapy protocol and the need for adjuvant radiotherapy in case of metastasis. Current NCCN guidelines recommends MRI and/or CT of region of disease, thorax CT, F-18 FDG-PET/CT and/or bone scan for evaluation of distant metastasis at initial staging of patients diagnosed with osteosarcoma [10]. F-18 FDG-PET/CT has been shown to have superior sensitivity and specificity than conventional radiological imaging in detecting distant metastasis (except lung) in primary bone tumors [4]. F-18 FDG-PET/CT and other conventional imaging modalities have close diagnostic accuracy in evaluation of the primary tumor in osteosarcoma; on the other hand, F-18 FDG-PET/CT has been shown to be superior in detecting lymphatic dissemination (95% versus 25%) and bone metastasis. Metastatic bone lesions not visible on bone scan can accurately be detected on F-18 FDG-PET/CT. Justly, thin-slice CT is superior to nonenhanced, nondiagnostic F-18 FDG-PET/CT imaging in detection of milimetric metastatic lung nodules. On the other hand, the CT component of hybrid imaging may enable detection of lung nodules even without FDG uptake [2,11]. In accordance with actual data, all nodules and metastatic findings on diagnostic chest CT were consistent with F-18 FDG-PET/CT results in all patients included in this research. In addition, 5 of 12 patients (41.6%) with lymphatic metastases were upstaged from localised disease to metastatic disease. F-18 FDG-PET/CT made a positive contribution to 2 patients treatment plan after detecting bone metastases of lytic character, while no pathologic findings were present on bone scan. F-18 FDG-PET/CT additionally detected findings consistent with intramedullary bone marrow metastasis in 6 patients further contributing to defining extent of disease. 

Current study data support that metabolic parameters from F-18 FDG-PET/CT imaging modality can be used to predict progression-free and overall survival. Despite the presence of multiple studies claiming metabolic parameters success in determining prognosis, to the best of our knowledge, there are no more studies proving the relation between these parameters and stage of disease at time of diagnosis. An increase in the level of SUVmax, MTV, and TLG is observed as the level of aggression and size of the primary tumor increases and an increased likelihood of detecting metastasis during staging of follow-up is present in these patients. The results of this study concluded that while there is a greater probability of existence of lung and/or lymphatic metastasis with high MTV and TLG values of the primary tumor in osteosarcoma patients, these parameters can not efficiently predict the presence of bone and/or bone marrow metastasis. This study also concludes that TLG is a more reliable prognostic marker than SUVmax in patients with lung and lymphatic metastasis. This can be explained by TLG being a parameter directly related to both the tumor volume and metabolic activity, also while SUVmax represents a very small area of the tumor, TLG represents a greater area of the tumor tissue hence providing metabolic data. 

It is known that osteosarcoma patients with a low TNR have a worse prognosis post neoadjuvant chemotherapy [12, 13]. In a study about the relationship between metabolic parameters SUVmax and TLG derived from staging F-18 FDG-PET/CT and TNR in patients receiving standard chemotherapy, Costelloe et al. stated that, TNR (above and below 90%) can be predicted by TLG but could not be predicted by SUVmax levels (P = 0.05, P = 0.62, respectively). High degree of TNR (above 90%) is seen in cases with lower TLG values [6]. This study also came to the conclusion that while SUVmax values do not predict TNR (P = 0.25), high TLG values are able to predict TNR (< 90% necrosis) (P = 0.027). Our study also concludes that MTV values can predict TNR, which is lower in patients with higher MTV values besides TLG (P = 0.012). Similarly, Davis et al. presented a study of 34 pediatric osteosarcoma patients and found that SUVmax values are not effective in predicting TNR but that SUVmax derived from interim F-18 FDG-PET/CT imaging on the 5th and 10th weeks of neoadjuvant chemotherapy can be more efficient in predicting TNR [14]. 

Current guidelines and studies recommend continuation of therapy if there is a positive response to therapy which means viable tumor cells are below 10% in operation material after extensive resection in both localised and metastatic osteosarcoma patients. On the other hand, in case of a poor response it is recommended to change the therapy possibly to a more aggressive regimen [12,13]. Deley et al. found a statistically significant increase in TNR when second line therapy regimens etoposide and ifosfamide where used in addition to methotrexate and doxorubicin in osteosarcoma therapy [15]. Accordingly, Goorin et al. showed that when therapy was changed to high doses of second line regimens such as ifosfamide and etoposide, an improvement was observed in therapy response parameters such as TNR and radiological response criteria. Of the 43 pediatric osteosarcoma patients included in this study, TNR was 100% in 4 patients, 90%–99% in 19 patients; therefore, 59% high histological response level was reached in patients in total [16]. 

Although second line/aggressive chemotherapy protocols managed an increase in TNR, not all patients can be candidates for this kind of therapy due to increase in toxic side effects. Therefore, it sould be important to decide which patients would be able to benefit from second line treatment after staging. In our study, by proving that lower levels of TNR can be foreseen in patients with higher metabolic parameters (MTV and TLG), and by using this parameters, we can confidently make the decision to change therapy protocols, which aim to increase TNR hence contributing to overall survival.

F-18 FDG-PET/CT is efficiently used in evaluating response to therapy in lymphoma and many solid tumors [17]. An interim F-18 FDG-PET/CT is possible to evaluate response to treatment early on in patients receiving one or two courses of neoadjuvant chemotherapy. Current NCCN guidelines and studies state that interim F-18 FDG-PET/CT is useful in both evaluation of response to therapy and in use as a prognostic marker of progression free and overall survival [18–20]. There has been an increase in research of the relationship between the ratio of change in SUVmax, MTV and TLG (staging and post-therapy values), TNR-histopathological response [6,21–24]. Also, the relation between change in metabolic parameters post-neoadjuvant chemotherapy and progression-free and overall survival are amongst new study areas [6,24]. In a study on 40 pediatric-adolescent osteosarcoma patients by Hawkins et al. higher TNR was found in patients with SUV2/SUV1 (change in SUVmax before and after chemotherapy) below 0.5 or post neoadjuvant chemotherapy SUVmax (SUV2) less than 2 mg/dL. Also, patients with high level TNR and SUV2 values lower that 2.5 mg/dL were reported as having longer progression free survival [24]. In a study by Im et al. with 14 patients, F-18 FDG-PET/CT was performed prior to neoadjuvant therapy, after one cycle of therapy (interim) and at end of therapy. MTV and TLG values at interim and end of therapy were compared with initial values and a positive correlation was found between the ratio of change of MTV and TLG and TNR. A high level TNR (90%–100%) has been shown in patients with SUVmax values below 3.0 mg/dL postchemotherapy with 100% sensitivity and 89% specifity [25]. Thus, F-18 FDG-PET/CT metabolic parameters from staging, interim, and response to therapy provide important prognostic information by predicting TNR and progression free and overall survival rates in osteosarcoma patients. 

The present study has several limitations. First, only staging F-18 FDG-PET/CT imaging and not interim and/or end of therapy F-18 FDG-PET/CT was available in our study group, which restricted our comparing ratio of change in metabolic parameters with predicting histopathological response. Second, histopathological verification was not performed to all metastatic findings. The decision was made based on clinical follow-up and additional imaging modalities. Finally, the patient inclusion criteria could have had a better standardisation if the study was prospective instead of retrospective. The issues we emphasize in our study need to be supported by prospective studies in larger series.

As a result, in pediatric osteosarcoma patients staging whole body F-18 FDG-PET/CT imaging has been shown to accurately distinguish metastatic and localised disease and effectively contribute to staging. In addition, it has shown that the metabolic parameters (MTV and TLG) of the primary tumor are important and accurate parameters used to predict postchemotherapy TNR. 

## Informed consent

The data in this application were used with the approval of the patients.
